# The Relationship between Social Capital and Self-Efficacy in Women with Gestational Diabetes Mellitus: A Cross Sectional Study

**DOI:** 10.4314/ejhs.v30i4.9

**Published:** 2020-07-01

**Authors:** Sedigheh Hasani Moghadam, Elham Yousefi Abdolmaleki, Fatemeh Alijani, Nastaran Bagherian Afrakoti, Jila Ganji

**Affiliations:** 1 Student Research Committee, Mazandaran University of Medical Sciences, Sari, Iran; 2 Department of Internal Medicine, Faculty of Medicine, Mazandaran University of Medical Sciences, Sari, Iran; 3 Department of Reproductive Health and Midwifery, School of Nursing and Midwifery, Mazandaran University of Medical Sciences, Sari, Iran; 4 Department of Reproductive Health and Midwifery, Sexual and Reproductive Health Research Center, Mazandaran University of Medical Science, Sari, Iran

**Keywords:** Self-Efficacy, Diabetes, Gestational, Social Capital

## Abstract

**Background:**

Self-efficacy is one of the most likely determinants of glucose self-management and self-monitoring by diabetic patients. Also, social capital is one of the effective social factors that may affect health behaviors. The aim of this study was to evaluate the relationship between social capital and self-efficacy in women with gestational diabetes mellitus (GDM).

**Methods:**

This descriptive-analytical cross-sectional study was conducted on 212 women with GDM in two diabetes center in Mazandaran, north of Iran, from April to July 2019. Patients' social capital and self-efficacy levels were measured using the Social Capital Questionnaire (SCQ) and Confidence in Diabetes Self-Care Scale questionnaire, respectively.

**Results:**

Among eight dimensions of social capital, the highest and the lowest mean scores were related to proactivity (21.3) and tolerance of diversity (5) dimensions. The mean (standard deviation=SD) of self-efficacy total score was 40.7(18.2), indicating moderate self-efficacy. Pearson correlation coefficient indicated that there was significant positive relationship between all dimensions of social capital and self-efficacy (p˂0.05). In addition, the results of multiple regression analysis indicated that community participation, neighborhood connections, family and friends' connections, tolerance of diversity and work connections, explained 55% of the variance in self-efficacy in women with GDM (p˂0.05).

**Conclusion:**

The results highlighted a significant positive relationship between social capital and self-efficacy in women with GDM. Improving women’s social capital may enhance their self-efficacy in controlling GDM.

## Introduction

Gestational diabetes mellitus (GDM) is the most common metabolic disorder encountered by women during their pregnancies that was first detected during pregnancy (1–5). Globally, the prevalence of GDM is growing (6), ranging from 1% to 14%, due to different diagnostic criteria (3,7). GDM imposes a risk for both the mother and the fetus (5,7). The negative outcomes of GDM includes preeclampsia, early cesarean and prematurity, macrosomia, shoulder dystocia or delivery injuries like facial paralysis, bone fracture, hypoglycemia, hospitalizing in neonatal intensive care unit, and increasing risk of developing type 2 diabetes mellitus for the mother in the first years after delivery (2,8). These patients should be carefully managed and monitored in terms of blood glucose level and life style modifications (1,4).

Self-efficacy is one of the most likely determinants of glucose self-management and self-monitoring by diabetic patients. Self-efficacy is one of the constructs of the Bandura social cognitive theory that explains the interaction between individual, behavioral and environmental factors in health and sickness (9–10). It is faith in self to conduct a special behavior successfully and expect positive results. Self-efficacy is an important prerequisite for changing behaviors (11). In addition, self-efficacy is a suitable framework for patient’s understanding, predicting and commitment to self-care in treating diabetes. Making life style changes, like dietary habits, smoking cessation and exercising, require high levels of self-confidence and self-efficacy (12). Social capital is another major factor that plays an important role in human health. Social capital has been defined as "social attributes", which include networks, norms, community and trust, as a facilitator for coordination between human capital and social capital (13). Social capital includes the norms of social systems which provide people participations in social actions to gain mutual benefits (14–16). Studies indicated that social capital has a protective effect on reducing stressful conditions, risky behaviors, depression, psychological disorders, mortality and improving general health (9,13). Also, it can promote healthy behavior in patients (17–18). It has been previously shown that there is a significant relationship between social capital and obesity and diabetes self-control (18–19). Also, it has been shown that social capital is inversely related to risky behaviors and their outcomes. Social capital can contribute to health-enhancing behaviors like exercising, healthy life style and reduce social isolation and social abnormalities (13,20). Despite the importance and potential role of social capital and self-efficacy in managing chronic diseases, like diabetes, there is a paucity of information about social capital and its relationship with self-efficacy among women with GDM. Therefore, the aim of this study was to evaluate the relationship between social capital and self-efficacy in women with GDM.

## Methods

This cross-sectional study was conducted on women with confirmed GDM who were referred to Ghaemshahr diabetes center and high-risk pregnancy clinic of Imam Khomeini Hospital in Sari, Northern Iran, from April to July 2019. After obtaining approval from the institutional Ethics Committee, the researcher referred to diabetes center and pregnancy clinic every day in order to check the referred patients. After obtaining written informed consent from patients who meet the inclusion criteria, using convenience sampling, 212 consecutive patients with GDM were enrolled in this study. The inclusion criteria were physician-confirmed diagnosis of GDM, 24–28 weeks of pregnancy, literacy, no history of drug addictions (such as psychotropic drugs, alcoholic drinks, cigarette and hookah), no history of any acute or chronic diseases such as diabetes, cardiovascular disease, respiratory disease, kidney disease, thyroid disorders, epilepsy or hypertension) and not having high risk pregnancies (preeclampsia, abnormal bleeding, placenta Previa and twin pregnancy). The exclusion criteria were occurrence of any pregnancy-related complications and experiencing a stressful event during the six month ago (serious illness of husband and children, death of a loved one, accidents, family dispute, divorce, immigration and financial bankruptcy) (3,21). The following questionnaires were used to collect the data.

### Socio-demographic and clinical questionnaire

This questionnaire was developed by the authors and includes some questions such as women’s age, pregnancy age, last fasting blood sugar and one-and two-hours postprandial glucose levels, educational level, husband’s job, women’s job, number of children, economic status and body mass index (BMI).

### Social Capital Questionnaire (SCQ)

This questionnaire was designed in 2000 by Onyx and Bullen. It consists of 36 questions and its main goal is measuring social capital from peoples' view point (22–23). The questionnaire includes 8 dimensions or subscales including value of life (3 items), tolerance of diversity (3 items), neighborhood connections (5 items), family and friends’ connections (3 items), work connections (4 items), community participation (7 items), feelings of trust and safety (5 items) and proactivity (6 items). Scoring the questionnaire is based on 4-point Likert scales as 1 (No, not at all), to 4 (Yes, definitely or nearly always). The highest score indicates the highest social capital (24–26). The reliability of this questionnaire was assessed and confirmed previously in Iran (Cronbach alpha=0.79) (24). Also, it had been indicated that the reliability of all dimensions of this questionnaire was above 0.70 by Intra-class correlation coefficient (16, 23).

### Confidence in Diabetes Self-Care Scale (CIDS)

CIDS is the questionnaire for measuring self-efficacy in patients provided by Van Der Van. This questionnaire includes 21 items for evaluating patients’ ability in self-monitoring. It was assessed by 5-option Likert scale ranging from zero to very high and the scores were from zero to 4. The lowest total score is zero, and the highest one is 84 (27).

### Sample size

The estimation of sample size was based on a presumed effect size of 0.1, a statistical power of 80%, and a type I error of 5%. The overall proper sample size was found to be 212 participants.

### Statistical analysis

The normality of data distribution was assessed by Skewness-Kurtosis test. Descriptive (frequency, mean, standard deviation [SD]) and inferential statistics (Pearson correlation, multiple regression) were used for data analysis, using IBM SPSS® version 25 software.

## Results

The findings indicated that the mean (SD) age of all women was 29.8 (6.8) years, and their mean (SD) gestational age was 26.3 (1.4) weeks. The mean (SD) fasting blood sugar level in women with GDM was 120 (25.5) mg/dl and one-and two-hours postprandial glucose level were 183.3

(25.2) and 151.7 (24.2) mg/dl, respectively. Most of the women (58%) and their husbands (61.3%) had university education. The majority of women (82.1%) and their husbands (50.9%) were housewives and self-employed, respectively. The majority of women had no children (61.8%); 22.6% of the women were dissatisfied with their economic status. According to the BMI on early pregnancy, the majority of women (52.8%) were over-weight (Table 1).

**Table 1:** Socio-demographic and clinical features in women with GDM.

Variable	Frequency (%)
**Women's Education Level**	
Illiterate	12(5.7)
Less than diploma	77(36.3)
Academic education	123 (58)
**Husband's Education Level**	
Illiterate	11(5.2)
Less than diploma	71(33.5)
Academic education	130(61.3)
**Women's Occupational Status**	
Housewife	174(82.1)
Employed	34(17.9)
**Husband's Occupational Status**	
Unemployed	22(10.4)
Employed	48(22.6)
Worker	108(50.9)
Others	34(16.1)
**Number of children**	
No child	131(61.8)
One child	57(26.9)
Two or more children	24(11.3)
**Economic status**	
Very satisfied	41(19.4)
Satisfied	36(17)
Relatively satisfied	45(21.2)
Dissatisfied	42(19.8)
Very dissatisfied	48(22.6)
**BMI**	
Thin (less than 19.8)	20(9.4)
Normal (19.8–25.9)	70(33)
Overweighed (26–29)	112(52.8)
Fat (more than 29)	10(4.8)

Among eight dimensions of social capital, the highest and the lowest mean scores were related to proactivity (19.3) and tolerance of diversity (5) dimensions. Also, the mean (SD) of the total self-efficacy score in women with GDM was 40.7(18.2), indicating moderate selfefficacy (Table 2).

**Table 2:** descriptive statistics of social capital dimensions and self-efficacy in women with GDM

Variables	Least	Highest	Average	SD
Value of life	3	10	5.5	1.7
Community participation	7	28	14.2	5.7
Proactivity	8	24	19.3	5.2
Feelings of trust and safety	5	19	12.1	2.9
Neighborhood connections	5	19	13.9	3.9
Family and friends' connections	3	12	9	2.6
Tolerance of diversity	3	10	5	1.7
Work connections	4	14	8.5	2.2
Self-efficacy	7	82	40.7	18.2

There is a significant positive relationship between all dimensions of social capital (value of life, tolerance of diversity, neighborhood connections, family and friends' connections, work connections, community participation, feelings of trust and safety and proactivity.) and self-efficacy (P<(0.05) (Table 3).

**Table 3:** Pearson correlation coefficient among dimensions of social capital and self-efficacy in women with GDM

Variables	(kh)	(S_8_)	(S_7_)	(S_6_)	(S_5_)	(S_4_)	(S_3_)	(S_2_)	(S_1_)
Self-efficacy (kh)									
Value of life(S_1_)	0.3**								-
Community participation (S_2_)	0.6**							-	0.3**
Proactivity (S_3_)	0.5**						-	0.4**	0.4**
Feelings of trust and safety (S_4_)	0.4**					-	0.6**	0.3**	0.4**
Neighborhood connections (S_5_)	0.4**				-	0.6**	0.7**	0.2**	0.4**
Family and friends connections (S_6_)	0.3**			-	0.5**	0.5**	0.5**	0.3**	0.4**
Tolerance of diversity (S_7_)	0.3**		-	0.4**	0.3**	0.3**	0.4**	0.2**	0.4**
Work connections (S_8_)	0.5**		0.3**	0.4**	0.4**	0.4**	0.6**	0.4**	0.4**

** P>0/001

The results of multiple regression analysis indicated that the variables, community participation, neighborhood connections, family and friends' connections, tolerance of diversity and work connections, predicted 55% of the variance in self-efficacy in women with GDM (p=0.05) (Table 4).

**Table 4:** Variance predictability of self-efficacy by variables of social capital dimensions in women with GDM

Variables	B	SE	Beta	t	P-value	R	R2	F
Self-efficacy	-	-	-	-	-	0.7	0.5	31.2
Value of life	0.1	0.6	0.01	0.2	0.7			
Community participation	1.4	0.1	0.4	8.3	<0.001			
Proactivity	0.4	0.2	0.1	1.4	0.1			
Feelings of trust and safety	0.2	0.4	0.04	0.6	0.5			
Neighborhood connections	0.9	0.3	0.2	2.6	0.009			
Family and friends' connections	-1.2	0.4	-0.1	-2.8	0.004			
Tolerance of diversity	1.3	0.5	0.1	2.2	0.02			
Work connections	1.1	0.5	0.1	2.2	0.02			

## Discussion

Social capital is considered as a factor that can positively affect the human health by mechanisms like easier access to community information, helping to decision making about health issues, social norms, increasing use of health services and providing mental support services (28). Some studies indicate a positive relationship between social capital and healthy lifestyle and health-promoting behaviors among patients with chronic diseases (29–30). The results of our study indicated that there were positive and significant relationships between all dimensions of social capital and self-efficacy. Additionally, the results of multiple regression analysis confirmed that some dimensions of social capital (community participation, neighborhood connections, family and friends' connections, tolerance of diversity and work connections) are effective factors on self-efficacy in women with GDM. Mizuno et al. in their cross-sectional study designed to evaluate the relationship between social capital and GDM showed that emotional support, trusting friends and neighbor interacting are significantly related to reducing diabetes prevalence (31). The results of a study by Nosrat Abadi et al. indicated the positive effects of social capital on mothers’ health status, which is consistent with the findings of the present study (32). Farajzadegan et al. evaluated the impact of social capital, as a forgotten topic, in diabetes monitoring and showed that social capital has a positive role in monitoring of diabetes (13). In addition, a study by Kim et al. with the aim to investigate the relationships between self-efficacy, social support, physical activities and BMI in women with GDM in USA, showed that self-efficacy is positively and strongly related to appropriate physical activity, education and nutritional status before childbirth. Also, a negative association was found between self-efficacy and smoking behavior. In addition, social support from family and friends was associated with higher scores for a healthy diet (20). It has been previously showed that enhancing social capital scopes in patients with diabetes leads to better control of blood glucose level (19,33). Diabetic patients with a cohesive family, social networks and extensive communication have significantly greater understanding of their individual capacity to prevent and manage their blood glucose levels (13,17). There is a relationship between the size and quality of a person's social network and their health status (34). Having a good relationship with family and friends makes a good sense in human and results in positive mental health and better decision making for monitoring diabetes (33,35). Therefore, communications and information can regulate patients’ activities and enable them to achieve better overall disease control (19,33). The results of our study showed that among the eight dimensions of social capital, the highest and the lowest mean scores were related to proactivity and tolerance of diversity, respectively. It has been shown that the highest score for proactivity is associated with greater self-efficacy in patients with diabetes (36). According to the importance of fetal health in pregnancy, this dimension has the highest score in this study. Previous studies in different populations in Iran showed that the workplace connections and community participation were the highest and the lowest dimensions of social capital in the participants which is inconsistent with the results of the present study (16,37–38). It seems that low tolerance of diversity may be related to cultural, religious and social differences in each area of the study. As the most participants of this study were housewives, it is justified to find low score for workplace connections. Inactivity of organizations and local communities is another reason for low levels of workplace connections. Another possible explanation for this is the lack of controlling confounding variables that may bias the results of the study. This study has some limitations. Non-random sampling is one the limitations that may reduce generalizing of the study results. Also, as this study relies on self- report questionnaire, it may be susceptible to reporting bias and leading to information bias. In conclusion, the results of this study highlighted the significant and positive relationship between social capital and self- efficacy in women with GDM. It seems that using appropriate modalities which can improve the women’s social capital could enhance their self-efficacy in controlling GDM and better monitoring of their disease.

## References

[1] Hjelm K, Berntorp K, Frid A, Åberg A, Apelqvist J (2008). Beliefs about health and illness in women managed for gestational diabetes in two organisations. Midwifery.

[2] Alayoub H, Curran S, Coffey M, Hatunic M, Higgins M (2018). Assessment of the effectiveness of group education on knowledge for women with newly diagnosed gestational diabetes. Ir J Med Sci.

[3] Yessoufou A, Moutairou K (2011). Maternal diabetes in pregnancy: early and long-term outcomes on the offspring and the concept of "metabolic memory". Exp Diabetes Res.

[4] Ferrara A, Hedderson MM, Albright CL, Ehrlich SF, Quesenberry CP, Peng T (2011). A pregnancy and postpartum lifestyle intervention in women with gestational diabetes mellitus reduces diabetes risk factors: a feasibility randomized control trial. Diabetes Care.

[5] Östlund I, Hanson U, Björklund A, Hjertberg R, Eva N, Nordlander E (2003:1). Maternal and fetal outcomes if gestational impaired glucose tolerance is not treated. Diabetes Care.

[6] Dabelea D, Snell-Bergeon JK, Hartsfield CL, Bischoff KJ, Hamman RF, McDuffie RS (2005:1). Increasing prevalence of gestational diabetes mellitus (GDM) over time and by birth cohort: Kaiser Permanente of Colorado GDM Screening Program. Diabetes Care.

[7] Canivell S, Gomis R (2014). Diagnosis and classification of autoimmune diabetes mellitus. Autoimmun Rev.

[8] Benhalima K, Minschart C, Ceulemans D, Bogaerts A, Van Der Schueren B, Mathieu C (2018). Screening and management of gestational diabetes mellitus after bariatric surgery. Nutrients.

[9] Williams DM, Rhodes RE (2016). The confounded self-efficacy construct: conceptual analysis and recommendations for future research. Health Psychol Rev.

[10] Lin X, Lu R, Guo L, Liu B (2019). Social capital and mental health in rural and urban China: a composite hypothesis approach. Int J Environ Res Public Health.

[11] Strecher VJ, DeVellis BM, Becker MH, Rosenstock IM (1986). The role of self-efficacy in achieving health behavior change. Health Educ Q.

[12] Massouh A, Skouri H, Cook P, Huijer HA, Khoury M, Meek P (2020). Self-care confidence mediates self-care maintenance and management in patients with heart failure. Heart Lung.

[13] Farajzadegan Z, Nazer S, Keyvanara M, Zamani A (2013). Social capital – a neglected issue in diabetes control: a cross-sectional survey in Iran. Health Soc Care Community.

[14] Kawachi I, Kennedy BP, Glass R (1999). Social capital and self-rated health: a contextual analysis. Am J Public Health.

[15] Poortinga W (2006). Social relations or social capital? Individual and community health effects of bonding social capital. Soc Sci Med.

[16] Karimi Ali Abadi P, Ahmadi M, Khalilian A R, Gnji J (2020). Relationship between Social Capital, Psychological Well-Being, and Quality of Life in Patients with Diabetes Mellitus. J Mazandaran Univ Med Sci.

[17] Kaiser B, Razurel C, Jeannot E (2013). Impact of health beliefs, social support and self-efficacy on physical activity and dietary habits during the post-partum period after gestational diabetes mellitus: study protocol. BMC Pregnancy Childbirth.

[18] Holtgrave D.R CR (2006). Is social capital a protective factor against obesity and diabetes? Findings from an exploratory study. Annals of Epidemiology.

[19] Long JA, Field S, Armstrong K, Chang VW, Metlay JP (2010). Social capital and glucose control. J Community Health.

[20] Kim C, McEwen LN, Kieffer EC, Herman WH, Piette JD (2008). Self-efficacy, social support, and associations with physical activity and body mass index among women with histories of gestational diabetes mellitus. Diabetes Educ.

[21] Kordi M, Asghar Poor N, Mazloom S R, Aklaghi F (2016). Prediction of self-care behaviors of women with gestational diabetes based on their belief in their ability (self-efficacy). J Obstet Gynecol.

[22] Onyx J, Bullen P (2000). Measuring social capital in five communities. J Appl Behav Sci.

[23] Moradian Sorkhkalaee M, Nedjat S, Saiepour N (2012). Social capital among medical Students of Tehran University of Medical Sciences in 2011. Razi Med Sci.

[24] Yari A, Nadrian H, Rashidian H, Nedjat S, Esmaeilnasab N, Doroudi R, et al (2014). Psychometric properties of the Persian version of Social Capital Questionnaire in Iran. Med J Islam Repub Iran.

[25] Rimaz S, Vesali S, Nedjat S, Asadi-Lari M (2015). A study on factors that drive variation in the levels of social capital among people living with HIV/AIDS in Iran. Glob J Health Sci.

[26] Baheiraei A, Bakouei F, Bakouei S, Eskandari N, Ahmari Tehran H (2015). Social capital as a determinant of self-rated health in women of reproductive age: a population-based study. Glob J Health Sci.

[27] Van Der Ven NC, Weinger K, YI J, Pouwer F, Ader H, Van Der Ploeg HM (2003). The confidence in diabetes self-care scale: Psychometric properties of a new measure of diabetes-specific self-efficacy in Dutch and US Patients with type 1 diabetes. Diabetes Care.

[28] Li C, Wu Q, Liang Z (2019). Effect of poverty on mental health of children in rural China: the mediating role of social capital. Appl Res Qual Life.

[29] Verheaghe PP Bp, Verheaghe M, Van De Putte B (2012). The association between network social capital and selfe related health. Pouring old wine in new bottles? Health Place.

[30] Baradari AG, Emami Zeydi A, Aarabi M, Ghafari R (2011). Metformin as an adjunct to insulin for glycemic control in patients with type 2 diabetes after CABG surgery: a randomized double blind clinical trial. Pak J Biol Sci.

[31] Mizuno S, Nishigori H, Sugiyama T, Takahashi F, Iwama N, Watanabe Z, et al (2016). Association between social capital and the prevalence of gestational diabetes mellitus: an interim report of the Japan environment and children’s study. Diabetes Res Clin Pract.

[32] Nosrat Abadi M (2015). Exploring the relationship between social capital ans social support with mothers helth in mothers referring to health centers of sijan city. A Structurul model. Journal of Urmia Nursing and Midwifery.

[33] Karimi Moonaghi H, Emami Zeydi A, Mirhaghi A (2016). Patient education among nurses: bringing evidence into clinical applicability in Iran. Invest Educ Enferm.

[34] McPherson KE, Kerr S, McGee E, Morgan A, Cheater FM, McLean J (2014). The association between social capital and mental health and behavioural problems in children and adolescents: an integrative systematic review. BMC Psychol.

[35] Howsepian BA, Merluzzi TV (2009). Religious beliefs, social support, self-efficacy and adjustment to cancer. Psychooncol.

[36] Keyvanara M, Afshari M, Dezfoulian E (2018). The Relationship between Social Capital and Quality of Life among Patients Referring to Diabetes Centers in Isfahan, Iran. J Diabetes Res.

[37] Eftekharian R, Sum S, Sahaf R, Fadaee RV (2016). Validity and reliability of the Persian version of Onyx Social Capital measurement in elderly people. Iranian Journal of Ageing.

[38] Esmaeili Shahmirzadi S, Tol A SR, Nikooseresht Z, Fard F (2013). The relationship between quality of life and social capital among health workers in medical and health network of Rey city in 2012. Razi J Med Sci.

